# Safety of Biologic and Immune-Modulating Agents in Breast Augmentation and Reconstruction

**DOI:** 10.1093/asjof/ojag138.004

**Published:** 2026-07-24

**Authors:** William M Tian, Aneeq S Chaudhry, Tarifa H Adam, Daniel A Rabin, Matthew Ramsey, Erin Kelley, Katherine Kozlowski, Marco F Ellis, Robert D Galiano

**Affiliations:** Division of Plastic & Reconstructive Surgery, Northwestern University Feinberg School of Medicine, Chicago, IL, USA; Division of Plastic & Reconstructive Surgery, Northwestern University Feinberg School of Medicine, Chicago, IL, USA; Division of Plastic & Reconstructive Surgery, Northwestern University Feinberg School of Medicine, Chicago, IL, USA; Division of Plastic & Reconstructive Surgery, Northwestern University Feinberg School of Medicine, Chicago, IL, USA; Division of Plastic & Reconstructive Surgery, Northwestern University Feinberg School of Medicine, Chicago, IL, USA; Division of Plastic & Reconstructive Surgery, Northwestern University Feinberg School of Medicine, Chicago, IL, USA; Division of Plastic and Reconstructive Surgery, University at Buffalo Jacobs School of Medicine and Biomedical Sciences, Buffalo, NY, USA; Division of Plastic & Reconstructive Surgery, Northwestern University Feinberg School of Medicine, Chicago, IL, USA; Division of Plastic & Reconstructive Surgery, Northwestern University Feinberg School of Medicine, Chicago, IL, USA

## Abstract

**Purpose:**

Autoimmune conditions such as psoriasis, rheumatoid arthritis, psoriatic arthritis, Crohn’s disease, ulcerative colitis, and systemic lupus erythematosus are increasingly treated with biologic and targeted immune-modulating agents. As more patients receiving these therapies pursue elective breast augmentation or implant-based breast reconstruction, surgeons face ongoing uncertainty regarding the potential impact of biologic exposure on postoperative wound healing, infection risk, seroma formation, and implant-related complications. Current evidence is limited, fragmented, and largely extrapolated from non-plastic surgery populations, leaving clinicians without clear data to guide perioperative decision-making or risk counseling. This study aims to evaluate whether preoperative exposure to biologic or immune-modulating therapies is associated with increased postoperative complications following breast augmentation and reconstruction procedures.

**Methods:**

A retrospective cohort study was conducted using the TriNetX electronic health record database. Patients who underwent breast augmentation and breast reconstruction procedures were identified using relevant CPT codes. Patients were stratified according to documented preoperative exposure to a biologic or immune-modulating agent within six months prior to surgery, including tumor necrosis factor-α (TNF-α) inhibitors, interleukin (IL) inhibitors, calcineurin inhibitors, sphingosine-1- phosphate (S1P) receptor modulators, Janus-associated kinase (JAK) inhibitors, mammalian target of rapamycin (mTOR) inhibitors, complement inhibitors, dihydroorotate dehydrogenase (DHODH) inhibitors, and B-cell targeting monoclonal antibodies. Propensity score matching (1:1) was performed to balance baseline demographic and clinical characteristics, including age, sex, race, body mass index, hypertension, hyperlipidemia, vitamin D deficiency, diabetes mellitus, immune disorders, cancer history, nicotine dependence, coagulation disorders, chronic obstructive pulmonary disease, peripheral artery disease, history of chemotherapy or radiation, and concurrent corticosteroid, antibiotic, or anticoagulant use.

Postoperative outcomes were assessed cumulatively at 30 days, 90 days, and 6 months following surgery. Evaluated complications included wound disruption (wound dehiscence or delayed healing), infection, seroma or hematoma, implant explantation or revision surgery, and unplanned return to the emergency department or hospital readmission. Risk ratios (RR) with 95% confidence intervals (CI) were calculated for each matched comparison, and statistical significance was defined as p < 0.05.

**Results:**

A total of 211,105 patients undergoing aesthetic and reconstructive breast surgery procedures were identified, including 4,024 patients with documented preoperative exposure to a biologic or immune-modulating agent and 207,081 controls. After 1:1 propensity score matching, 3,980 patients remained in each cohort with balanced demographic and clinical characteristics. Across all postoperative intervals, biologic-exposed patients demonstrated complication rates comparable to matched controls. At 30 days, rates of wound dehiscence (1.51% vs. 1.44%), infection (3.00% vs. 2.84%), seroma/hematoma (1.91% vs. 1.78%), explant or revision surgery (1.21% vs. 0.91%), and return to the ED or readmission (2.25% vs. 3.02%) showed no significant differences (all p>0.05). Findings remained consistent at 90 days, with similar rates of wound dehiscence (3.31% vs. 3.02%), infection (5.72% vs. 5.08%), seroma/hematoma (3.24% vs. 2.97%), explant or revision surgery (2.66% vs. 2.32%), and ED visits/readmissions (3.91% vs. 4.47%, all p>0.05). At 6 months, complication rates continued to be comparable between cohorts, including wound dehiscence (3.91% vs. 3.69%), infection (7.09% vs. 6.35%), seroma/hematoma (3.92% vs. 3.64%), explant or revision surgery (6.52% vs. 5.84%), and ED visits/readmissions (5.27% vs. 6.18%), with all differences remaining statistically nonsignificant (p>0.05). Overall, biologic exposure was not associated with increased postoperative morbidity at any timepoint.

**Conclusion:**

Preoperative exposure to biologic or immune-modulating agents was not linked with higher rates of postoperative wound complications, infection, explantation, or readmission following breast augmentation and reconstruction procedures. Across the 30-day, 90-day, and 6-month time points, biologic-exposed patients had outcomes that were comparable to matched controls, suggesting that biologic treatment alone does not increase short-term surgical risk. These findings may help support more confident perioperative counseling and may prevent unnecessary interruption of essential diseasemodifying treatments. While the study demonstrates associations rather than causation, further prospective and mechanistic research is needed to clarify how these medications influence wound healing and to refine perioperative management strategies.

